# Effects of pore interconnectivity on bone regeneration in carbonate apatite blocks

**DOI:** 10.1093/rb/rbac010

**Published:** 2022-02-16

**Authors:** Maab Elsheikh, Ryo Kishida, Koichiro Hayashi, Akira Tsuchiya, Masaya Shimabukuro, Kunio Ishikawa

**Affiliations:** Department of Biomaterials, Faculty of Dental Science, Kyushu University, 3-1-1 Maidashi Higashi-ku, Fukuoka 812-8582, Japan

**Keywords:** bone substitutes, carbonate apatite, pore interconnectivity, bone regeneration

## Abstract

Porous architecture in bone substitutes, notably the interconnectivity of pores, is a critical factor for bone ingrowth. However, controlling the pore interconnectivity while maintaining the microarchitecture has not yet been achieved using conventional methods, such as sintering. Herein, we fabricated a porous block using the crystal growth of calcium sulfate dihydrate, and controlled the pore interconnectivity by limiting the region of crystal growth. The calcium sulfate dihydrate blocks were transformed to bone apatite, carbonate apatite (CO_3_Ap) through dissolution–precipitation reactions. Thus, CO_3_Ap blocks with 15% and 30% interconnected pore volumes were obtained while maintaining the microarchitecture: they were designated as CO_3_Ap-15 and CO_3_Ap-30, respectively. At 4 weeks after implantation in a rabbit femur defect, new bone formed throughout CO_3_Ap-30, whereas little bone was formed in the center region of CO_3_Ap-15. At 12 weeks after implantation, a large portion of CO_3_Ap-30 was replaced with new bone and the boundary with the host bone became blurred. In contrast, CO_3_Ap-15 remained in the defect and the boundary with the host bone was still clear. Thus, the interconnected pores promote bone ingrowth, followed by replacement of the material with new bone. These findings provide a useful guide for designing bone substitutes for rapid bone regeneration.

**Figure:**
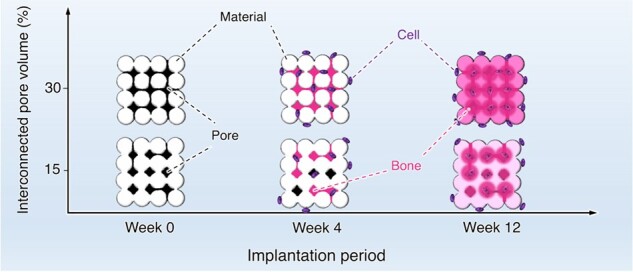

## Introduction

Bone defects resulting from severe trauma or wide resection of bone tumors require a graft material for their reconstruction [1–4]. Autografts using the iliac crest, fibula or resected bone after devitalization have been used as an effective option [1, 2, 4]. Although autografting is still regarded as the gold standard for bone reconstruction in post-traumatic conditions [1–3], problems, such as limited tissue mass availability, risk of infection and increased morbidity of the donor site, are unavoidable [5–7]. Therefore, the development of effective bone-substitute materials is urgently needed.

The outcome of bone reconstruction is primarily determined by the chemical composition of the grafting material [8, 9]. Among the bone-substitute materials currently used, carbonate apatite (CO_3_Ap), which is the main component of the bone mineral [10–12], has recently attracted much attention owing to its ability to be replaced by new bone during the bone remodeling process, as observed in autografts [13–15]. A much higher osteoconductivity of CO_3_Ap was also reported as compared with those of sintered hydroxyapatite (HAp) and β-tricalcium phosphate [16–22].

The architecture of the bone substitutes is also a factor that significantly affects the outcome of bone regeneration [23]. In particular, porous architectures that increase their specific surface area and vascular permeability are considered essential for the success of reconstruction [23–25]. Although the pore size and volume of the materials typically affect bone ingrowth and formation [26–32], the pore geometry, such as uniformity, orientation and interconnectivity, is also a key design consideration. Notably, an interconnected architecture of pores has been considered effective for the penetration of body fluids and cells, contributing to bone ingrowth. Although the introduction of interconnected pores [2, 23, 33–35] and/or continuous channels [29, 30, 36–40] has been achieved, controlling the pore interconnectivity while maintaining the microarchitecture has not been realized by conventional methods, such as sintering.

Interconnected porous blocks can be fabricated by interconnecting the granules and creating gaps between them. However, in the case of CO_3_Ap, the direct interconnection of CO_3_Ap granules is difficult because they have no setting ability and sinterability below the decomposition temperature. Therefore, we previously fabricated interconnected porous CO_3_Ap blocks by interconnecting precursor granules followed by compositional transformation to CO_3_Ap through a dissolution–precipitation reaction; they were interconnected by a thermal fusion with an organic binder [41], the hydrate expansion of CaO granules [42], the setting reaction of α-tricalcium phosphate spheres [43] and the setting reaction of calcium sulfate hemihydrate (CaSO_4_·1/2H_2_O) granules [44]. The setting reaction of CaSO_4_·1/2H_2_O granules with water occurs with highly aggressive crystal growth of calcium sulfate dihydrate (CaSO_4_·2H_2_O). Although this crystal growth easily causes clogging of gaps between granules and spoiling of the pore interconnectivity of CaSO_4_·2H_2_O, we found that this clogging could be avoided by regulating the amount of water [44]. In other words, the pore interconnectivity of the CO_3_Ap blocks might be adjustable based on this water removal process.

In this study, we aimed to fabricate CO_3_Ap blocks with different pore interconnectivities while maintaining the microarchitecture for histological evaluation. The pore interconnectivity was controlled by limiting the region of crystal growth of CaSO_4_·2H_2_O during the setting reaction. Centrifugal force was used to partially remove water and limit the region of crystal growth. After converting the CaSO_4_·2H_2_O blocks to CO_3_Ap through dissolution–precipitation reactions, we implanted the CO_3_Ap blocks in the rabbit femur defect to evaluate the influence of pore interconnectivity on bone ingrowth, new bone formation and material resorption.

## Materials and methods

### Fabrication of CO_3_Ap block

In our previous study, a porous CO_3_Ap block was fabricated through dissolution–precipitation reactions of a porous calcium sulfate anhydrous (CaSO_4_) block, which was obtained by the setting reaction of CaSO_4_·1/2H_2_O granules with water followed by dehydration heat treatment [44]. In this study, we fabricated CO_3_Ap blocks with different pore interconnectivities based on this method, with slight modifications.

In brief, commercial CaSO_4_·1/2H_2_O powder (NEW FUJIROCK WHITE, improved dental stone powder, GC Corporation, Tokyo, Japan) was mixed with distilled water at a liquid to powder ratio of 0.2 cm^3^·g^−1^ to obtain a set CaSO_4_·2H_2_O block, followed by granulating into the size of 100–200 µm and dehydrating at 120°C for 24 h. The CaSO_4_·1/2H_2_O granules obtained were placed in an acrylic mold (φ 6 mm × 9 mm) with covered stainless steel meshes. Two wetting conditions were used to control the pore interconnectivity: the packed granules were immersed in distilled water for 30 s followed by centrifugation at a speed of 500× *g* for 3 min using a tabletop centrifuge (Model 2420, Kubota, Tokyo, Japan), or immersed in distilled water for 30 s without centrifugation. This centrifuge process was designed to remove water except for that retained by interfacial tension with the granules. Both samples were kept inside the mold at room temperature for 24 h to prepare porous CaSO_4_·2H_2_O blocks. The blocks obtained were heat-treated at a heating rate of 1°C·min^−1^–750°C and maintained at 750°C for 6 h. After cooling to room temperature, the blocks were immersed in a 1 mol·dm^−3^ solution of Na_2_CO_3_ (Fuji Film Wako Pure Chemical Corporation, Osaka, Japan) at 60°C for 3 days to convert CaSO_4_ into calcium carbonate (CaCO_3_). The CaCO_3_ blocks obtained were immersed in a 0.1 mol·dm^−3^ solution of Na_2_HPO_4_ (Fuji Film Wako Pure Chemical Corporation) at 60°C for compositional transformation to CO_3_Ap. The CO_3_Ap blocks obtained from the CaSO_4_·2H_2_O block centrifuged at 500× *g* for water removal and those without centrifugation had 30% and 15% interconnected pore volumes, respectively, as shown in the Results and discussion section. Hence, the CO_3_Ap blocks derived from the CaSO_4_·2H_2_O block centrifuged at 500× *g* and those without centrifugation are referred to as CO_3_Ap-30 and CO_3_Ap-15, respectively. The compositional transformation from CaCO_3_ to CO_3_Ap in a Na_2_HPO_4_ solution was conducted for 7 (CO_3_Ap-30) or 14 days (CO_3_Ap-15).

### Characterization

The crystal phases of the CO_3_Ap blocks were analyzed by powder X-ray diffraction (XRD; D8 Advance diffractometer, Bruker AXS GmbH, Karlsruhe, Germany) with CuKα radiation generated at an acceleration voltage of 40 kV and a current of 40 mA. The chemical characteristics of the CO_3_Ap blocks were analyzed by Fourier transform infrared (FTIR) spectroscopy (FT/IR-6200, JASCO, Tokyo, Japan) using the KBr disk method. The carbonate content in the CO_3_Ap blocks was determined by performing the elemental carbon–hydrogen–nitrogen (CHN) analysis (MT-6, Yanako Analytical Instruments, Kyoto, Japan). The remaining sulfate content in the CO_3_Ap blocks was assessed by inductively coupled plasma–optical emission spectrometry (ICP-OES, Optima 7300 DV, PerkinElmer, MA, USA).

The surface morphologies of the CO_3_Ap blocks were investigated by scanning electron microscopy (SEM; S-3400N, Hitachi High Technologies, Tokyo, Japan) with an acceleration voltage of 10 kV, after being coated with gold-palladium using a magnetron sputtering machine (MSP-1S, Vacuum Device Co., Ibaraki, Japan). Elemental analysis on the fractured surface of the samples was performed by energy-dispersive X-ray (EDX) spectroscopy coupled with the SEM with an accelerating voltage of 15 kV. The volume and size distribution of the interconnected pores in the CO_3_Ap blocks were analyzed using mercury intrusion porosimetry (AutoPore 9420, Shimadzu Corporation, Kyoto, Japan). The internal morphology of the CO_3_Ap blocks was investigated using the micro-computed tomography (μ-CT) scanning (ScanXmate-L090T, Comscan, Kanagawa, Japan).

The penetrability of the CO_3_Ap blocks was evaluated by the infiltration of a 0.05% toluidine blue solution (Fuji Film Wako Pure Chemical Corporation). The block was then immersed in the dye solution for 3 min. The dyed blocks were dried at 60°C for 1 h.

The mechanical strength of the CO_3_Ap blocks was evaluated in terms of the compressive strength and diametral tensile strength. After the diameter and height of each block were measured with a micrometer (IP65, Mitutoyo Co., Ltd, Kanagawa, Japan), the block was crushed by a loaded force at a crosshead speed of 1 mm/min using a universal testing machine (Autograph, AGS-J, Shimadzu Corporation). Eight samples per group were analyzed to determine the average compressive strength and diametral tensile strength. The total porosity of the CO_3_Ap blocks was determined from their bulk density (determined from each block’s weight and volume) and calculated using the theoretical density of HAp (3.16 g·cm^−3^). The apparent porosity of the CO_3_Ap blocks was determined according to Archimedes’ principle using water as the immersion liquid. Eight samples per group were analyzed to determine the average total porosity and apparent porosity.

The release profiles of calcium and phosphorus were investigated by immersing 100 mm^3^ of the CO_3_Ap blocks in 5000 mm^3^ of 4-(2-hydroxyethyl)-1-piperazineethanesulfonic acid (HEPES) buffer solution (Fuji Film Wako Pure Chemical Corporation) at 37°C. The amount of calcium and phosphorus released was determined by ICP-OES using Optima 7300 DV (PerkinElmer). The HEPES buffer solution was replaced with equal volume of fresh solution every seventh day.

### *In vitro* evaluation

As described in our previous work [45], MC3T3-E1 cells (Riken BioResource Center, Ibaraki, Japan) were maintained in a cell culture medium, which was an alpha modification of Eagle’s minimum essential medium (α-MEM; Gibco, USA) supplemented with 10% fetal bovine serum (Gibco), 100 U·cm^−3^ penicillin, 100 mg·cm^−3^ streptomycin and 0.25 mg·cm^−3^ amphotericin B (Gibco). The cells were incubated at 37°C in a humidified atmosphere containing 5% CO_2_. For sample preparation for cell invasion assay, each CO_3_Ap block (φ 6 mm × 3 mm) was tightly inserted into the edge of an acrylic tube (φ 6 mm × 4 mm) to inhibit free migration of cells along the side of the block. The CO_3_Ap blocks inserted in the tubes were placed in a well plate so that one side of the block and tube were in contact with the bottom of the plate. All samples were sterilized in 70% ethanol and rinsed with phosphate buffered saline (PBS). All sterilized samples were immersed in the medium for 1 day to prevent the burst release of calcium ions.

In cell invasion assay, MC3T3-E1 cells were seeded on the samples at an approximate initial concentration of 5.5 × 10^4^ cells·cm^−3^. After 7 days of incubation, the cells attached to the samples were rinsed with PBS and fixed with 4% paraformaldehyde for 15 min. For SEM observation, the cells were dehydrated in a graded ethanol series consisting of 50%, 60%, 70%, 80%, 90% and 99.5% ethanol, and dried by hexamethyldisilazane (Fuji Film Wako Pure Chemical Corporation) before being coated with gold–palladium. For fluorescence staining, the attached cells were permeabilized by immersing the samples in 0.5% Triton X-100 for 15 min. The non-specific reactions were blocked by 60 min of immersion in PBS containing 3% bovine serum albumin at room temperature. F-actins and nuclei of the attached cells were stained with Acti-stain 555 phalloidin (Cytoskeleton Inc., Denver, USA) and Hoechst 33342 (Dojindo, Kumamoto, Japan), respectively. Fluorescence images were captured from the bottom of the samples.

### *In vivo* evaluation

The animal experiments conducted in this study were approved by the Animal Care and Use Committee of Kyushu University (No. A30-293-0; issued 26 November 2018). Eighteen week-old male Japanese white rabbits (Japan SLC, Inc., Shizuoka, Japan) weighing 3.0–3.5 kg were utilized for the experiments. These rabbits were housed at the Center of Biomedical Research, Research Center for Human Disease Modeling, Graduate School of Medical Sciences, Kyushu University. They were maintained on a standard feed and water.

For sample implantation, the rabbits were initially anesthetized via intraperitoneal injection of a mixture of ketamine (30 mg·kg^−1^) and xylazine (5.0 mg·kg^−1^). The rear limbs were shaved to remove the fur, and then the skin was disinfected with 10% povidone-iodine (Meiji Seika Pharma Co., Ltd, Tokyo, Japan). The distal epiphysis of the femur was selected as the implant site because of its sufficient bone volume. An incision was made in the skin and periosteum of the femur, and a cylindrical defect (φ 6 mm × 3 mm) was created using a trephine bur. This defect size was adopted based on previous studies [40, 42], in which an effective comparison of material architecture of CO_3_Ap was made within 12 weeks without perforating into bone marrow region. It can be noted that this defect size is not generally regarded critical and may not be used for long-term experiments. CO_3_Ap-30 or CO_3_Ap-15 was implanted into the bone defect, and the periosteum and skin flap were closed (*n *=* *3 per group). The rabbits were allowed unrestrained movement in their cages after they recovery from anesthesia. After implantation for 4 and 12 weeks, the rabbits were sacrificed, and the specimens were removed.

The μ-CT images of the specimens were obtained using a μ-CT scanner (SkyScan, Bruker Corporation, MA, USA). For histomorphometric analyses, the specimens were fixed in 10% formalin neutral buffer solution, decalcified with 0.5 mol·dm^−3^ ethylenediaminetetraacetic acid, and embedded in paraffin. The paraffin blocks were sliced at 3-μm thickness using a sliding microtome (REM-710/CBUH, Yamato Kohki, Asaka, Japan). After deparaffinization, hematoxylin and eosin (HE) staining was performed using a general protocol.

### Statistical analysis

Statistical analyses were performed by Tukey’s test after one-way ANOVA using R software (The R Foundation for Statistical Computing, ver. 4.0.0, 2020). *P *<* *0.05 was considered statistically significant.

## Results and discussion

### Fabrication and characterization of CO_3_Ap blocks

**Figure 1.**
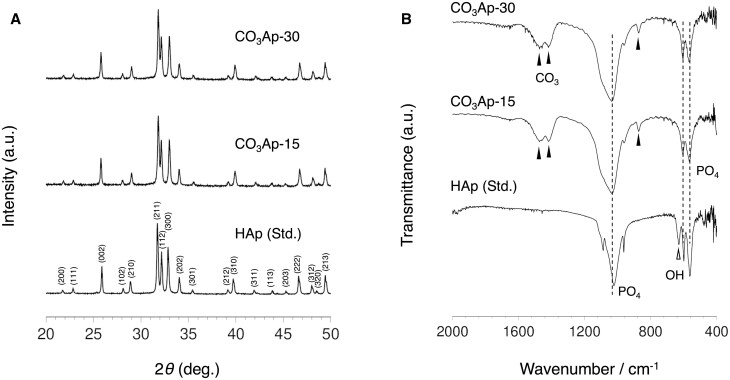
(**A**) XRD patterns and (**B**) FTIR spectra of CO_3_Ap-30, CO_3_Ap-15 and standard HAp. The miller indices of HAp were assigned from PDF# 00-009-0432.

The CO_3_Ap blocks derived from the CaSO_4_·2H_2_O blocks centrifuged at 500× *g* for water removal during the setting reaction (CO_3_Ap-30) and those without centrifugation processes (CO_3_Ap-15) showed the same XRD patterns, confirming that both were purely composed of apatite without remaining precursors, such as CaSO_4_ and CaCO_3_ (Fig. 1A). The remaining sulfate groups were not detected from ICP-OES. Furthermore, the FTIR spectra confirmed the presence of carbonate groups and the absence of hydroxyl groups (Fig. 1B). The absorption peaks detected at 1470 and 1420 cm^−1^ correspond to the substitution of hydroxyl and phosphate groups by carbonate groups (Fig. 1B). Therefore, the apatites obtained were both AB-type CO_3_Ap. The CHN analyses showed no significant differences in carbonate content between CO_3_Ap-30 and CO_3_Ap-15 (Table 1). The carbonate content is equal to that of human bone apatite (6–9 wt%) [12–14].

**Table 1.** Carbonate content of CO_3_Ap blocks

Sample	Carbonate content (wt%)
CO_3_Ap-30	7.5 ± 0.5
CO_3_Ap-15	7.2 ± 0.2

**Figure 2.**
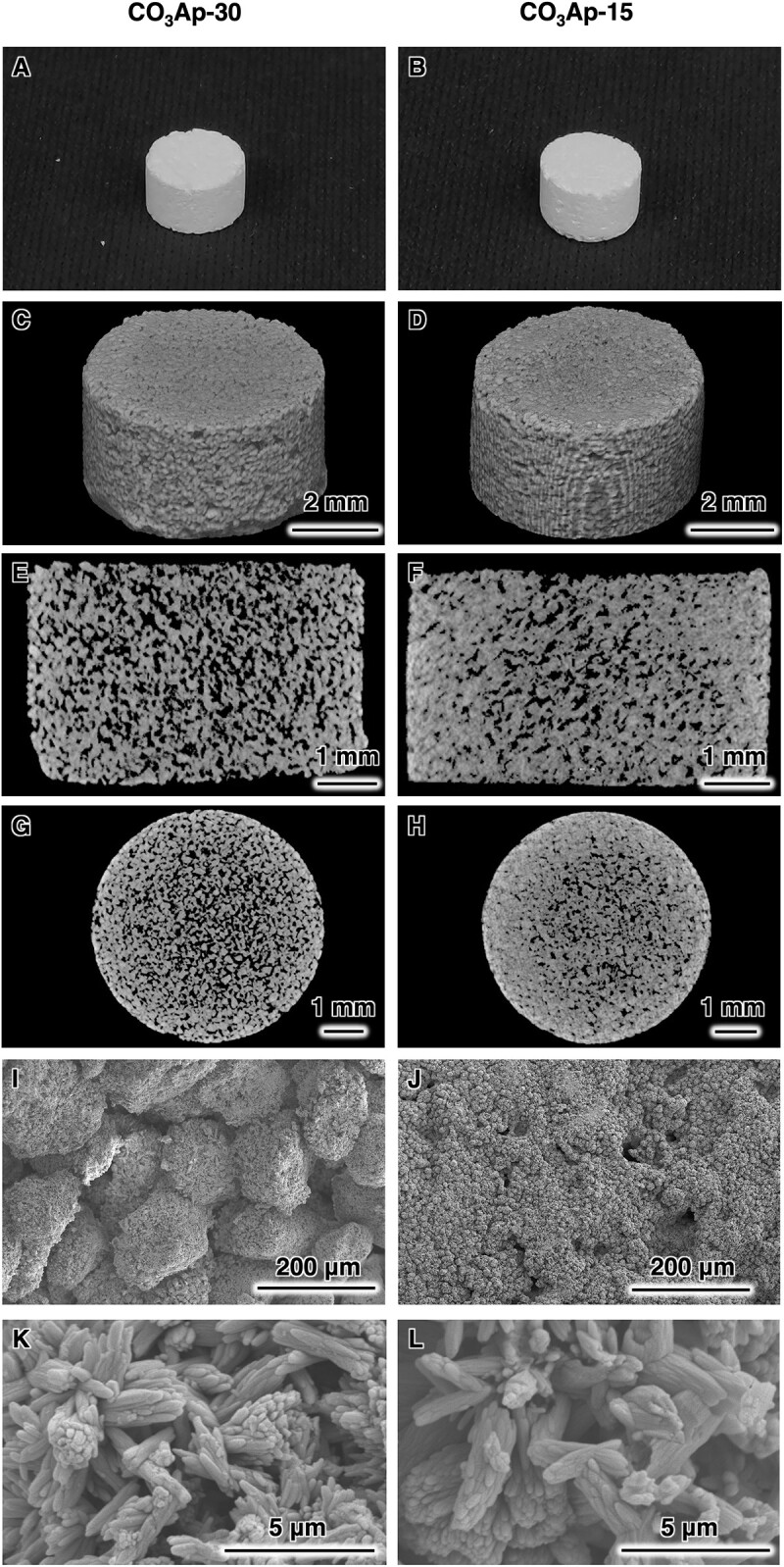
Photograph images of (**A**) CO_3_Ap-30 and (**B**) CO_3_Ap-15. μ-CT scanning observations of materials. μ-CT-based 3D reconstruction images of (**C**) CO_3_Ap-30 and (**D**) CO_3_Ap-15. μ-CT-based sliced images at a sagittal section of (**E**) CO_3_Ap-30 and (**F**) CO_3_Ap-15. μ-CT-based sliced images at a transverse section of (**G**) CO_3_Ap-30 and (**H**) CO_3_Ap-15. SEM images of (**I**) CO_3_Ap-30 and (**J**) CO_3_Ap-15. Magnified SEM images of (**K**) CO_3_Ap-30 and (**L**) CO_3_Ap-15.

Both CO_3_Ap blocks were cylindrical blocks constructed of granules (Fig. 2A–D). The 2D-sliced images showed a significant difference in the internal architecture (Fig. 2E–H). CO_3_Ap-30 showed a pore-interconnected structure throughout the sliced images (Fig. 2E and G). In contrast, CO_3_Ap-15 showed less continuous pore interconnections (Fig. 2F and H). The SEM images of the two groups showed a significant difference in surface morphology (Fig. 2I and J). The surface of CO_3_Ap-30 exhibited a clear bridging structure of granules (Fig. 2I), while the bridging pattern was smeared out on the surface of CO_3_Ap-15 (Fig. 2J). Both CO_3_Ap blocks were composed of rod-like crystals (Fig. 2K and L). The surface morphologies observed were consistent with those of CaSO_4_·2H_2_O blocks, which showed a similar pattern of granular bridging depending on the water removal process (Supplementary Fig. S1). Therefore, the multi-step compositional transformation of the blocks from CaSO_4_·2H_2_O to CO_3_Ap did not alter the macroscopic morphology. The structural difference between CO_3_Ap-30 and CO_3_Ap-15 is likely to reflect the difference in the crystal growth of CaSO_4_·2H_2_O. Although the crystal growth of CaSO_4_·2H_2_O causes expansion in general, free expansion was restricted at the area contacting with the wall of the mold used for filling CaSO_4_·1/2H_2_O granules. This restricted expansion resulted in a densified structure at the periphery of CaSO_4_·2H_2_O blocks and CO_3_Ap blocks (Fig. 2E–H), as also confirmed by a porosimetry along the diametral direction based on μ-CT images (Supplementary Fig. S2). This densification was more significantly observed in CO_3_Ap-15, where much crystal growth occurred. Despite this porosity gradient, the composition of both CO_3_Ap blocks was uniform from the outer edge to the central region, as confirmed by EDX analysis (Supplementary Fig. S3).

**Figure 3.**
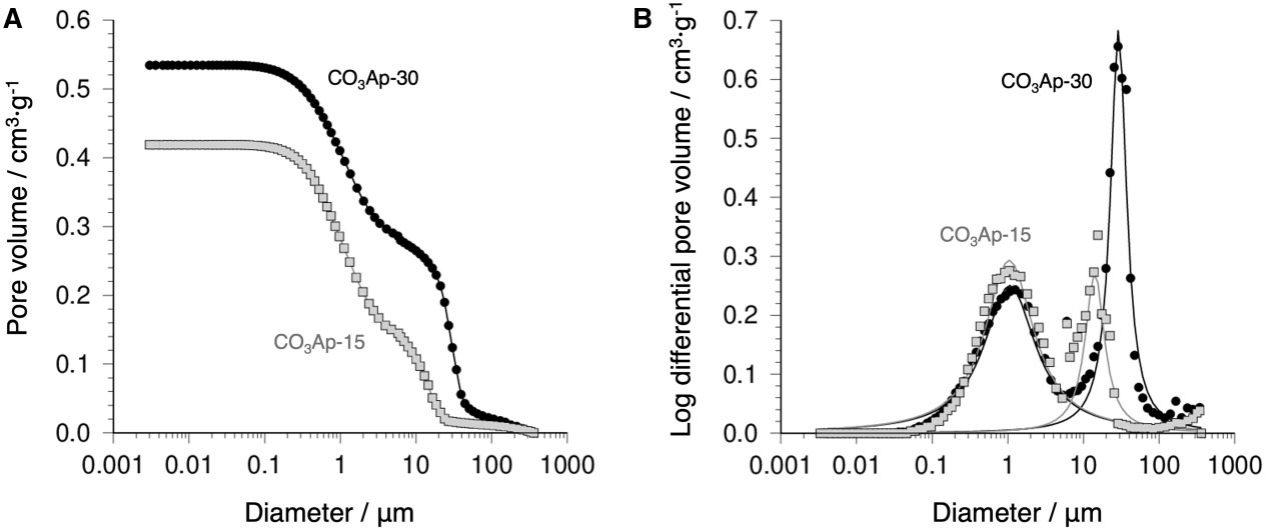
(**A**) Cumulative and (**B**) differential pore volume plotted as a function of pore diameter of CO_3_Ap-30 and CO_3_Ap-15. Differential pattern was best-fitted by two Lorentzian functions.

To find quantitative differences in the interconnected porous architecture between CO_3_Ap-30 and CO_3_Ap-15, mercury intrusion porosimetry was employed (Fig. 3). CO_3_Ap-30 showed a larger interconnected pore volume than CO_3_Ap-15 (Fig. 3A). Both the CO_3_Ap blocks had a pore size distribution at two different scales, namely, larger pores of ∼29 μm (CO_3_Ap-30) or 14 μm (CO_3_Ap-15) and smaller pores of ∼1 μm in diameter (Fig. 3B). These larger and smaller pores are likely to originate in the intergranular and intercrystalline gaps of CO_3_Ap, respectively. By computing the peak areas of larger pores, the interconnected pore volumes in CO_3_Ap-30 and CO_3_Ap-15 were determined as 280 and 130 mm^3^·g^−1^, respectively. These pore volumes (280 and 130 mm^3^·g^−1^) correspond to 30% and 15% of the total volume, respectively.

**Figure 4.**
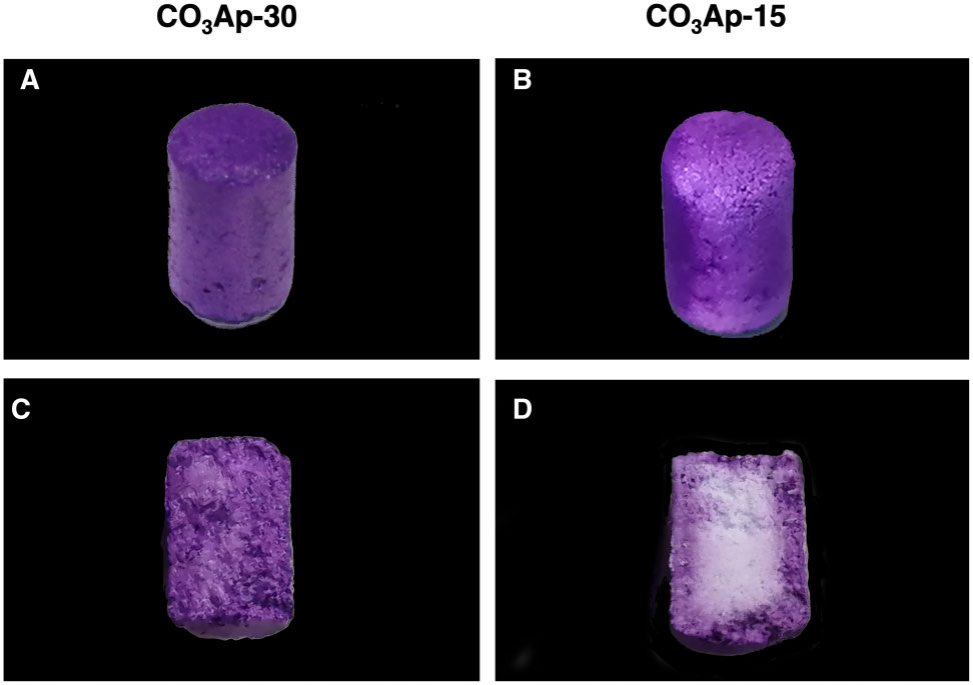
Photograph images of materials after dye penetration assay. Perspective views of (**A**) CO_3_Ap-30 and (**B**) CO_3_Ap-15. Cross-sectional views of (**C**) CO_3_Ap-30 and (**D**) CO_3_Ap-15.

Pore interconnectivity was visualized using a dye penetration assay (Fig. 4). Complete dye penetration was observed in CO_3_Ap-30 showing a stained cross-section, whereas the assay of CO_3_Ap-15 resulted in a limited stained area, indicating full and partial pore interconnectivity of CO_3_Ap-30 and CO_3_Ap-15, respectively. This observation coincides with our previous study, in which the water removal process was identified as the key to creating interconnected pores [44].

**Figure 5.**
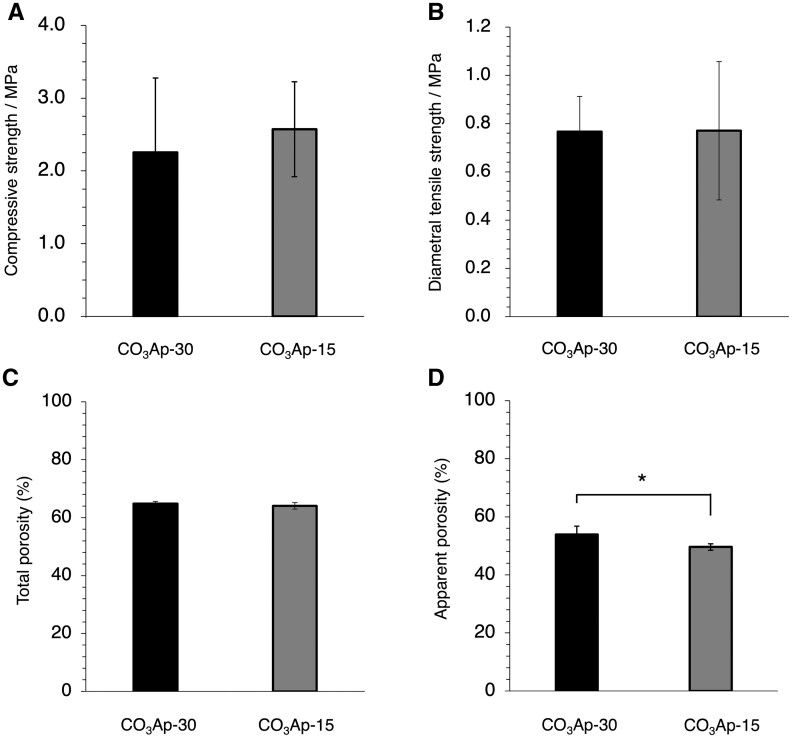
(**A**) Compressive strength, (**B**) diametral tensile strength, (**C**) total porosity and (**D**) apparent porosity of CO_3_Ap-30 and CO_3_Ap-15. *P <* *0.05.

There was no significant difference in the mechanical strength and total porosity between CO_3_Ap-30 and CO_3_Ap-15 (Fig. 5A–C). In other words, the improved pore interconnectivity did not affect the mechanical strength in accordance with the maintained total porosity. Since the equal amount of the starting substance, CaSO_4_·1/2H_2_O granules were used, both CO_3_Ap-30 and CO_3_Ap-15 were obtained from the same amount of calcium, resulting in the equal total porosity. In contrast, the apparent porosity of CO_3_Ap-30 determined by the Archimedes method was significantly higher than that of CO_3_Ap-15, confirming the increased interconnected pores and the reduced closed pores (Fig. 5D). At present, the mechanism necessary to yield an equal mechanical strength while having different pore interconnectivity has not been fully clarified. The mechanical strength is thought to depend mainly on the interlocking degree of CO_3_Ap crystals. Although CO_3_Ap-30 had a fractured surface with clear granules-fused structure, CO_3_Ap-15 exhibited a more disordered fractured surface (Supplementary Fig. S3). Therefore, the CO_3_Ap crystals may be also interlocked at the interstitial gaps between granules as well as the intergranular regions, when the crystal growth is prominent. These two contributions to the mechanical strength may result in an equal mechanical strength with an equal total porosity.

**Figure 6.**
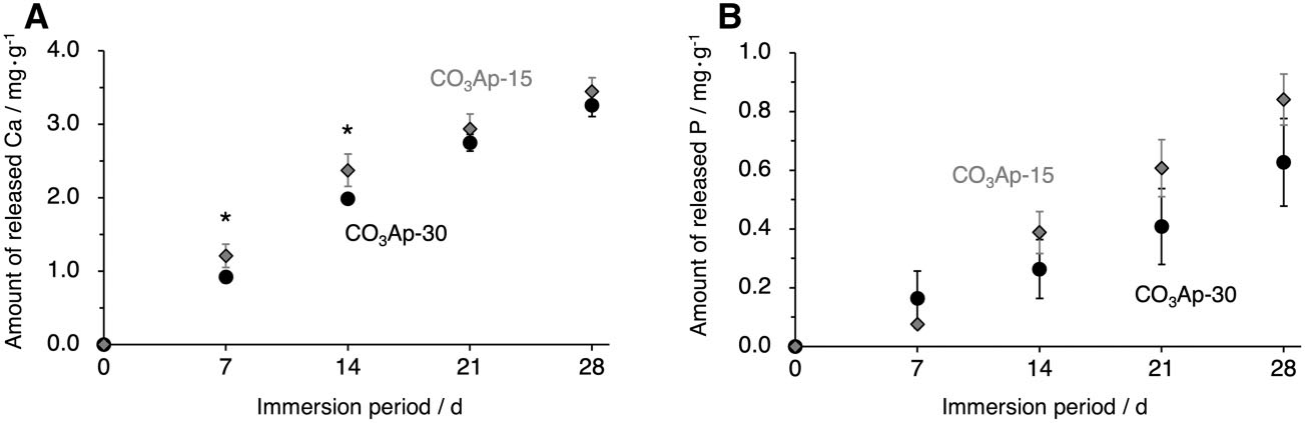
The amount of (**A**) calcium and (**B**) phosphorus released from CO_3_Ap-30 (black circles) and CO_3_Ap-15 (gray diamonds) to the HEPES buffer during 28 days of immersion measured using ICP-OES. *P <* *0.05.

**Figure 7.**
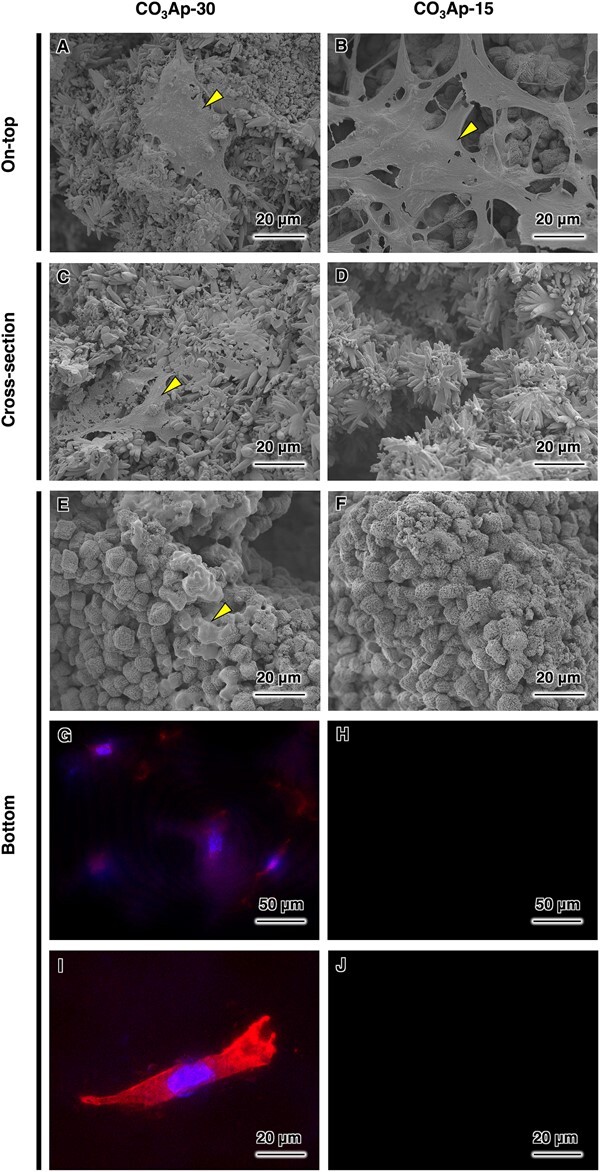
Cell invasion assay of the samples with 3-mm thickness for 7 days of culture period. SEM images at the cell-seeded on-top surface of (**A**) CO_3_Ap-30 and (**B**) CO_3_Ap-15. SEM images at a cross-section of (**C**) CO_3_Ap-30 and (**D**) CO_3_Ap-15. SEM images at the bottom surface of (**E**) CO_3_Ap-30 and (**F**) CO_3_Ap-15. F-actins (red) and nuclei (blue) of attached cells on the bottom surface of (**G**) CO_3_Ap-30 and (**H**) CO_3_Ap-15. Magnified fluorescence images of the bottom surface of (**I**) CO_3_Ap-30 and (**J**) CO_3_Ap-15. Yellow triangles indicate cells.

The *in vitro* behaviors of the CO_3_Ap blocks were investigated in terms of ion release and cell invasion. During the 28 days of immersion in HEPES buffer, both CO_3_Ap-30 and CO_3_Ap-15 tended to constantly release calcium and phosphate ions at nearly equivalent rates (Fig. 6). This is consistent with the nearly equal specific surface areas, which were estimated from mercury intrusion porosimetry (CO_3_Ap-30: 1.79 m^2^·g^−1^; CO_3_Ap-15: 1.73 m^2^·g^−1^). After 7 days of cell culture, the cells tended to grow preferentially on the top of CO_3_Ap-15 surface rather than CO_3_Ap-30 surface (Fig. 7A and B). In contrast, no cells were found inside and on the bottom of CO_3_Ap-15, while CO_3_Ap-30 allowed cells to invade to a depth of 3 mm (Fig. 7C–J). Therefore, the difference in pore interconnectivity affected penetration of cells as well as body fluid.

### *In vivo* evaluation

The above characterization of CO_3_Ap-30 and CO_3_Ap-15 demonstrated that only the pore interconnectivity was distinctly different between the two groups, whereas the chemical composition, micropore distribution, mechanical strength and total porosity were equal. Therefore, the comparison between CO_3_Ap-30 and CO_3_Ap-15 provides an effective evaluation of the effects associated with pore interconnectivity. Thus, both the CO_3_Ap blocks were implanted in a defect in the rabbit femur.

**Figure 8.**
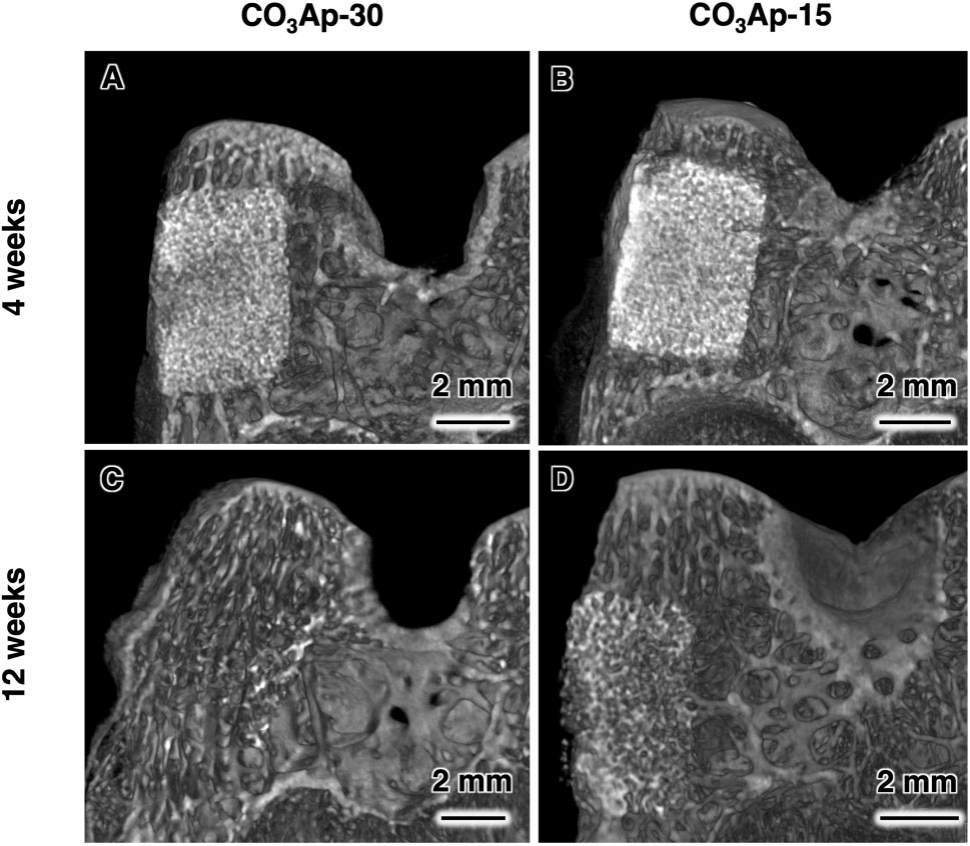
μ-CT images of rabbit femurs with defects reconstructed by grafting CO_3_Ap-30 at (**A**) 4 weeks and (**C**) 12 weeks after implantation, and CO_3_Ap-15 at (**B**) 4 weeks and (**D**) 12 weeks after implantation

The μ-CT images showed that at 4 weeks after implantation, CO_3_Ap-30 and CO_3_Ap-15 remained in the bone defects and maintained their clear boundary with the host bone (Fig. 8A and B). CO_3_Ap-30 had a lower CT-positive value in the bone defect and a lower contrast with the host bone. At 12 weeks after implantation, the radiopacity of the defect area decreased in both groups, suggesting that the resorption of CO_3_Ap was progressed (Fig. 8C and D). This was accompanied by a diminished dense pattern of implant material, which had been observed during the 4-week period. In the case of CO_3_Ap-30 at the 12-week period, the CT-positive value in the defect was fairly close to that of the host bone (Fig. 8C). In other words, CO_3_Ap-30 had the potential to be resorbed and replaced by cancellous new bone in 12 weeks. In contrast, the remaining material area of CO_3_Ap-15 was clearly observed even at the 12-week period (Fig. 8D). Therefore, CO_3_Ap-15 was resorbed and replaced by new bone at a slower rate.

**Figure 9.**
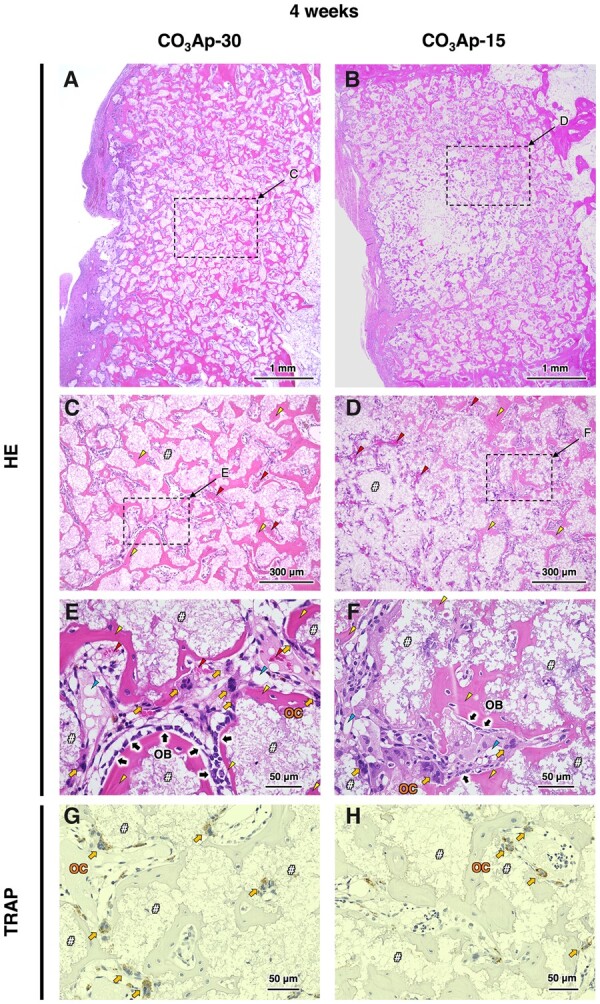
HE-Stained histological images of rabbit femurs with defects reconstructed by grafting (**A**) CO_3_Ap-30 and (**B**) CO_3_Ap-15 at 4 weeks after implantation. (**C**) and (**D**) show magnified versions of the images presented in (A) and (B), respectively. (**E**) and (**F**) show higher magnification images of typical regions in (A) and (B), respectively. (**G**) and (**H**) show TRAP-stained images in the region near (E) and (F), respectively. Yellow triangles, red triangles, blue triangles, ‘OB’, ‘OC’ and ‘#’denote new bone, red blood cells, adipose tissues, osteoblasts, osteoclasts and material, respectively.

**Figure 10.**
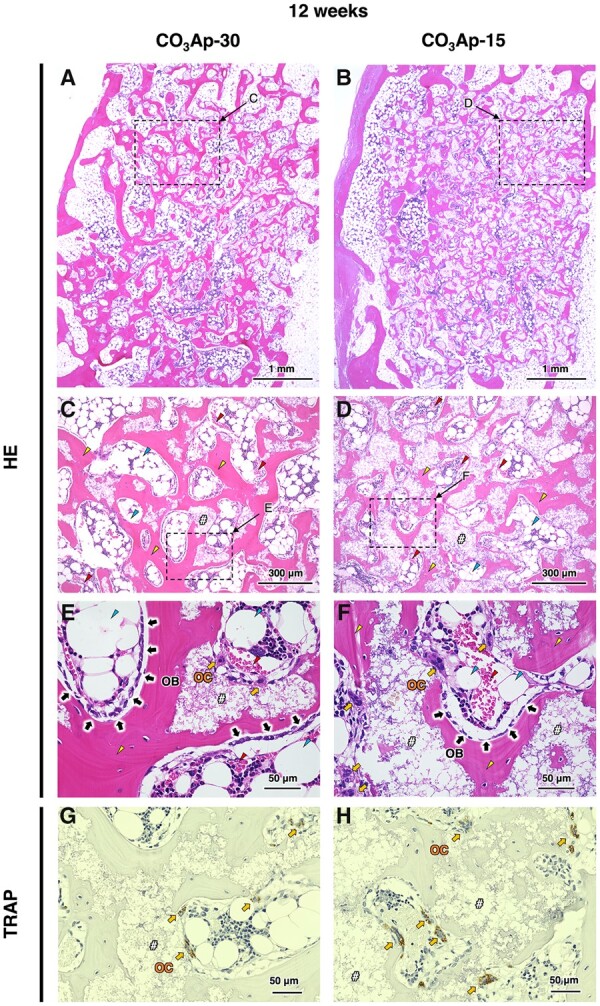
HE-Stained histological images of rabbit femurs with defects reconstructed by grafting (**A**) CO_3_Ap-30 and (**B**) CO_3_Ap-15 at 12 weeks after implantation. (**C**) and (**D**) show magnified versions of the images presented in (A) and (B), respectively. (**E**) and (**F**) show higher magnification images of typical regions in (A) and (B), respectively. (**G**) and (**H**) show TRAP-stained images in the region near (E) and (F), respectively. Yellow triangles, red triangles, blue triangles, ‘OB’, ‘OC’ and ‘#’denote new bone, red blood cells, adipose tissues, osteoblasts, osteoclasts and material, respectively.

To investigate new bone formation, bone ingrowth and material resorption, histological analysis was performed with HE staining. At 4 weeks after implantation, both samples were continuously attached to the host bone without any fibrous tissue, confirming their histocompatibility and osteoconductivity (Fig. 9A and B). Moreover, new bone formed over the entire area inside CO_3_Ap-30, whereas the bone area inside CO_3_Ap-15 was limited to the region near the host bone. This indicates that interconnected pores in CO_3_Ap were easily occupied by new bone due to the absence of barriers for bone ingrowth, while closed pores prevent bone ingrowth into the center region of the material. Osteoblasts, osteoclasts and red blood cells were distributed inside the materials, indicating that the pores were effective for allowing cellular migration and providing an osteoconductive environment (Fig. 9C–H). Even in the area of limited bone formation in CO_3_Ap-15 (i.e. the center region), a large number of cells resided, which is thought to be due to the resorption of material by opening the pores that were initially not interconnected (Fig. 9D). Osteoblasts were aligned along the edge of the new bone, while osteoclasts were distributed around the material surfaces (Fig. 9E–H). At 12 weeks after implantation, CO_3_Ap-30 and CO_3_Ap-15 showed progressed resorption, as manifested in the reduced area of their building granules (Fig. 10A and B). As a morphological change, the CO_3_Ap-30-implanted area exhibited a trabecular bone-like pattern consisting of new bone and adipose tissue (Fig. 10A). The boundary between the CO_3_Ap-30-implanted area and the surrounding host bone became blurred, while the CO_3_Ap-15-implanted area was still clearly distinct from the host bone (Fig. 10A and B). Therefore, CO_3_Ap-30 was mostly replaced by cancellous-structured new bone in 12 weeks. In both groups, bone marrow-like tissue as well as adipose tissue was also observed (Fig. 10C and D). Therefore, migration or generation of soft tissue as well as hard tissue became pronounced upon progressed resorption of the material. These soft tissues may have migrated to the bone defect through the bone remodeling. Furthermore, mesenchymal and hematopoietic stem cells, which were thought to have differentiated mainly into osteoblasts and osteoclasts at the 4-week period, may have switched to adipocytes and myelocytes, respectively, to a certain extent. Osteoblasts in an array were observed at the interface between the bone and adipose tissue, while osteoclasts were mainly found near the remaining materials (Fig. 10E–H). Thus, the bone remodeling process governed by the osteoblastic new bone formation and osteoclastic resorption proceeded continuously during the implantation period.

**Figure 11.**
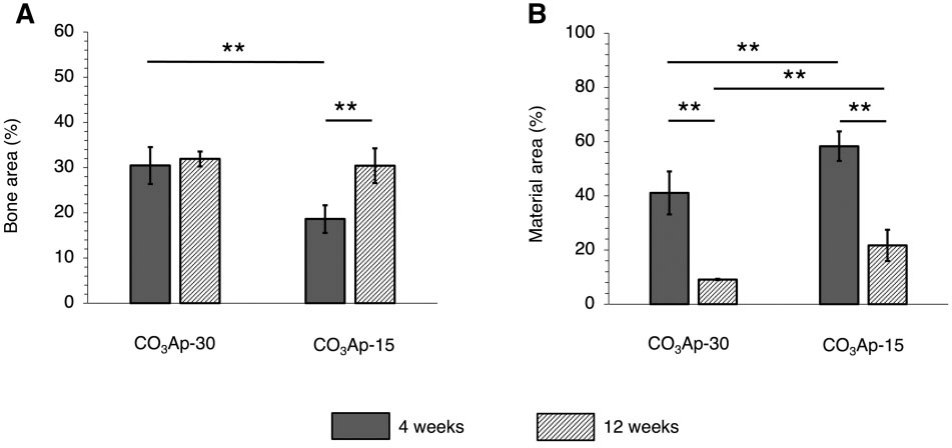
Area percentages of (**A**) bone and (**B**) remaining material in each bone defect. **P <* *0.01.

The area percentages of bone and material in the bone defect were calculated from the histological sections (Fig. 11A and B). At 4 weeks after implantation, the bone area inside CO_3_Ap-30 was significantly larger than that inside CO_3_Ap-15 (Fig. 11A), corresponding to the observed difference in new bone formation in the region distant from the host bone (Fig. 9A and B). From Weeks 4–12, both groups showed an increase in the bone area, although this was not statistically significant in the case of CO_3_Ap-30 (Fig. 11A). Moreover, the material areas drastically decreased at 12 weeks after implantation (Fig. 11B), corresponding to the rapid resorption of CO_3_Ap- and replacement by cancellous-structured new bone (Figs 9 and 10).

The above *in vivo* evaluation clearly demonstrates that the interconnectivity of pores has a remarkable influence on new bone formation and material resorption, or CO_3_Ap-to-bone remodeling. The pore size of the CO_3_Ap blocks used in this study was smaller than that typically adopted. Considering that the pores smaller than 50 µm are usually referred to as micropores [32], both CO_3_Ap-30 and CO_3_Ap-15, which have larger pores distributed around 29 and 14 μm, respectively, should be referred to as microporous CO_3_Ap blocks. Nevertheless, these pores were liquid-penetrable (Fig. 4) and could be occupied by cells through bone ingrowth (Figs 9 and 10). Therefore, although quite small, the pores distributed around 29 and 14 μm acted as a penetrable space for bony tissue, similar to macropores. Typically, the effects of macropore size have been associated with angiogenesis, which is in competition with the ingrowth of fibrous tissue [28, 30]. Correspondingly, the osteoconductivity evaluated by the bone area decreased below a certain threshold of pore size (∼500 μm) [27]. From a geometrical point of view, osteoconductivity should also be affected by pore geometry, even at the same pore size and volume. For example, the lower threshold of the effective pore size was decreased to ∼300 μm by using a uniformly oriented honeycomb CO_3_Ap blocks [29, 30]. In this study, we used a CO_3_Ap block with fully interconnected pores smaller than 100 μm in diameter, and achieved almost complete replacement of the implanted material with new bone in 12 weeks (Fig. 10A, C and E). Therefore, 3D porous architectures with high pore interconnectivity are associated with rapid bone regeneration, which may drastically shorten hospitalization. Furthermore, upon significant resorption of the material, bone marrow-like tissues were observed at the 12-week period (Fig. 10). A detailed mechanism is currently not available. Similarly, a previous investigation using honeycombed CO_3_Ap reported the generation of bone marrow-like tissue upon progressed resorption of CO_3_Ap with a high micropore volume, which might be associated with an elevated local Ca^2+^ concentration [46]. As shown in the porosimetry results, both the CO_3_Ap blocks prepared in this study had a micropore volume of more than 50% of the pore volume (Fig. 3). Considering that even interconnected pores were smaller than 100 μm, the overall volume occupied by micropores was extremely large. Therefore, further studies are needed to understand the tissue reactions that generate bone marrow-like tissues using highly microporous architectures.

## Conclusions

The CO_3_Ap blocks with 15% and 30% interconnected pore volumes, i.e. CO_3_Ap-15 and CO_3_Ap-30, for histological evaluation of the rabbit femur defect were fabricated in this study. Other structural parameters, such as microporosity, micropore size and total porosity, were equal between CO_3_Ap-15 and CO_3_Ap-30. The fabrication of CO_3_Ap-30 was achieved by a centrifuge method to limit the region of crystal growth of CaSO_4_·2H_2_O, followed by compositional transformation to CO_3_Ap. At the 4-week period, new bone formed over the entire region of CO_3_Ap-30, whereas a much more scarce formation of bone in the center region of CO_3_Ap-15 was observed. Therefore, the rate of bone ingrowth was significantly affected by the pore interconnectivity. At the 12-week period, the original block morphology of CO_3_Ap-30 mostly vanished upon replacement by cancellous-structured new bone, whereas CO_3_Ap-15 remained in the bone defect. From this observation, we conclude that introducing interconnected pores is a highly effective approach for realizing rapid bone regeneration by allowing earlier bone ingrowth, followed by replacement of the material with new bone.

## Supplementary data

Supplementary data are available at *REGBIO* online.

## Funding

This research was supported, in part, by Japan Agency for Medical Research and Development under Grant Number JP20im0502004, Grant-in-Aid for Research Activity start-up (18H06295) and Grant-in-Aid for Early-Career Scientists (20K18576) from Japan Society for the Promotion of Science.

## Author contributions

M.E. carried out the preparation and characterization of materials, participated in the design of the study and analyzed *in vivo* experimental results; R.K. wrote the manuscript, conducted *in vitro* and *in vivo* experiments, carried out the characterization of materials and supervised the study; K.H. critically reviewed the manuscript and participated in the design of the study; A.T. participated in the design of the study; M.S. conducted *in vitro* experiments and critically reviewed the manuscript; K.I. conceived and designed the experiments, critically reviewed the manuscript and supervised the study.

*Conflict of interest statement*. None declared.

## Supplementary Material

rbac010_Supplementary_Data
