# Supplementary material for: Effects of pore interconnectivity on bone regeneration in carbonate apatite blocks
Source: Regen Biomater. 2022 Feb 16;9:rbac010. doi: 10.1093/rb/rbac010 (PMC9017375; doi:10.1093/rb/rbac010)
Supplement: rbac010_Supplementary_Data [file rbac010_supplementary_data.docx]

**Supplementary Materials**

Effects of Pore Interconnectivity on Bone Regeneration in Carbonate Apatite Blocks

*Maab Elsheikh, Ryo Kishida*, Koichiro Hayashi, Akira Tsuchiya, Masaya Shimabukuro,*

*Kunio Ishikawa*

AUTHOR ADDRESS

Department of Biomaterials, Faculty of Dental Science, Kyushu University, 3-1-1 Maidashi Higashi-ku, Fukuoka 812-8582, Japan.

*Correspondence: kishida@dent.kyushu-u.ac.jp


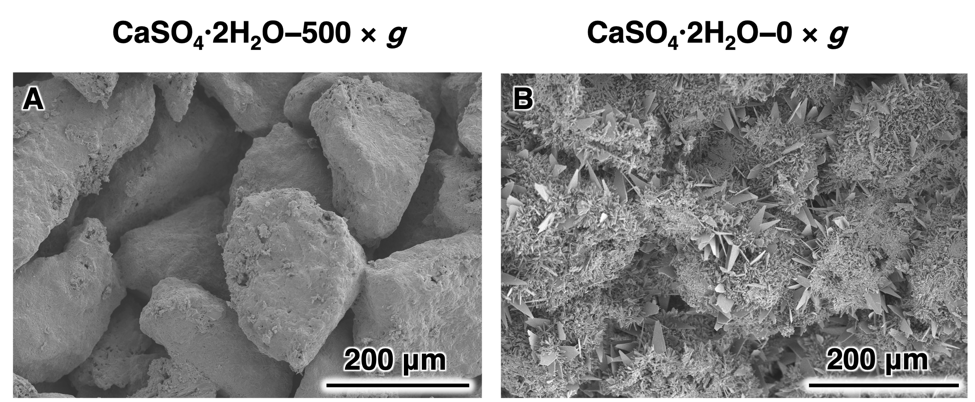
Fig. S1 SEM images of A) CaSO_4_·2H_2_O blocks centrifuged at 500 × *g* for water removal during the setting reaction (CaSO_4_·2H_2_O−500 × *g*) and B) those obtained without water removal processes (CaSO_4_·2H_2_O−0 × *g*).


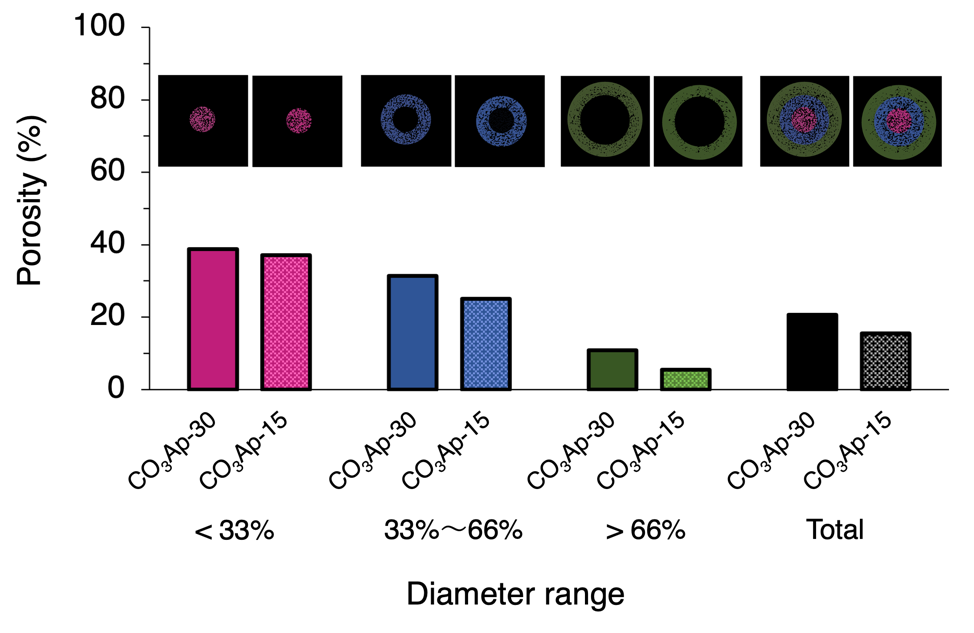


Fig. S2 μ-CT-based porosimetry at three different diameter ranges (< 33%, 33~66%, and > 66% of the original diameter) of CO_3_Ap-30 and CO_3_Ap-15.


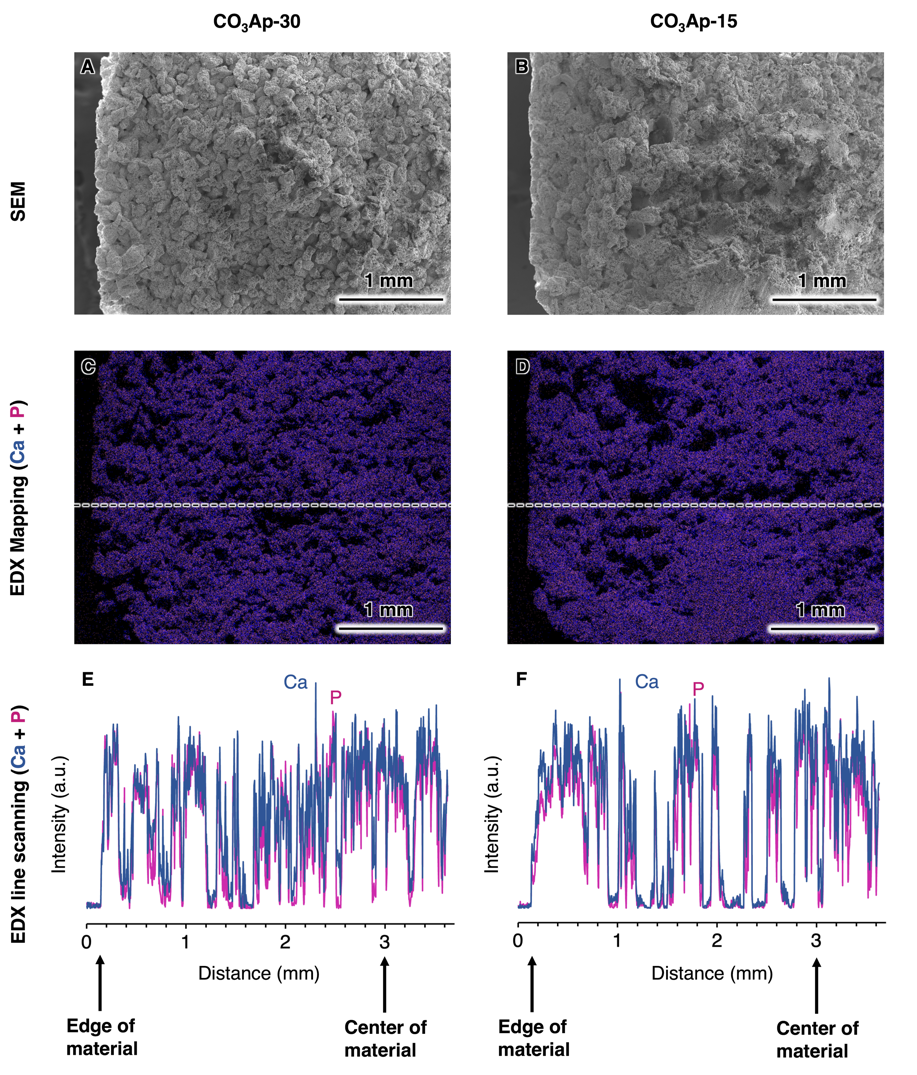
Fig. S3 EDX analysis for elemental distribution on the fractured surfaces. SEM images of A) CO_3_Ap-30 and B) CO_3_Ap-15. EDX mapping images of C) CO_3_Ap-30 and D) CO_3_Ap-15. EDX line scanning plot of E) CO_3_Ap-30 and F) CO_3_Ap-15. Blue and magenta indicates Ca and P, respectively.
